# Obturator Nerve Block for Postoperative Pain Control After Total Knee Arthroplasty: Case Series and Literature Review

**DOI:** 10.7759/cureus.92399

**Published:** 2025-09-15

**Authors:** Thierry Claude Bagaphou, Pierfrancesco Fusco, Fabrizio Fattorini, Fabio Gori, Domenico Pietro Santonastaso

**Affiliations:** 1 Anesthesiology and Critical Care, Città di Castello Hospital, Città di Castello, ITA; 2 Anesthesia and Intensive Care Unit, San Filippo and Nicola Hospital, Avezzano, ITA; 3 Anesthesiology, San Sebastiano Hospital, Rome, ITA; 4 Anesthesiology, Critical Care, Medicine and Pain Therapy, Sapienza University of Rome, Rome, ITA; 5 Anesthesia, Intensive Care and Pain Medicine, Santa Maria della Misericordia Hospital, Perugia, ITA; 6 Anesthesia and Intensive Care, AUSL (Azienda Unità Sanitaria Locale) Romagna M. Bufalini Hospital, Cesena, ITA

**Keywords:** adductor nerve block, ipack block, knee arthroplasty, obturator nerve block, postoperative pain control

## Abstract

For postoperative analgesia following total knee arthroplasty, the adductor canal block and the injection of local anesthetic between the popliteal artery and the posterior capsule of the knee (IPACK) are among the most frequently utilized regional anesthetic techniques. The adductor canal block specifically targets the anterior knee compartment, while the IPACK technique aims to block the sensory innervation to the posterior knee, thereby providing effective pain control. These interventions are commonly employed to minimize opioid consumption and improve overall pain management outcomes in the immediate postoperative period. According to the existing literature, the obturator nerve block is less commonly employed compared to other peripheral nerve blocks. The obturator nerve, a mixed nerve, plays a role in the innervation of the knee in conjunction with the femoral and sciatic motor nerves. In the present study, three patients who underwent total knee arthroplasty under subarachnoid anesthesia, supplemented with an adductor canal block for postoperative analgesia, reported moderate-to-severe pain in the immediate postoperative period. Subsequent effective analgesia was achieved through the administration of a single-shot obturator nerve block. The outcomes were highly favorable, demonstrating a significant improvement in pain control. The patients experienced adequate analgesia, thereby illustrating the efficacy of the obturator nerve block in managing pain following total knee arthroplasty, without impairing ambulation, and facilitating the early initiation of rehabilitation therapy. Based on our experience, the obturator nerve block is an effective analgesic technique that can be employed either in combination with other peripheral nerve blocks or as a rescue strategy when patients, despite having undergone other regional analgesia techniques, continue to report moderate-to-severe pain in the immediate postoperative period following total knee arthroplasty. In contrast to the femoral nerve block, which induces a broad motor blockade and substantially impairs ambulation, the obturator nerve block may cause motor impairment limited to thigh adduction, which is unlikely to significantly affect the patient’s ability to ambulate.

## Introduction

The obturator nerve is a mixed nerve that provides motor and sensory innervation. It originates from the L2, L3, and L4 spinal roots of the lumbar plexus. After its exit from the obturator foramen, it descends into the medial compartment of the thigh and divides into the anterior and the posterior terminal branches. The anterior branch runs downward between the adductor longus and brevis muscles, anastomoses with the saphenous nerve, innervates the intermedial region of the thigh, and participates in anteromedial innervation of the knee joint. The posterior branch descends between the adductor brevis and adductor major muscles and, together with branches of the sciatic nerve, participates in the innervation of the posterior part of the knee [1].

For the management of postoperative pain following knee surgery, femoral nerve block (either single-shot or continuous) and the combination of femoral and sciatic nerve blocks have been identified as among the most effective analgesic strategies. These techniques are frequently supplemented by other modalities such as the genicular nerve block and newer as the infiltration between the popliteal artery and the posterior capsule of the knee (IPACK) block [2].

In terms of postoperative complications, the fascia iliaca nerve block, femoral nerve block, sciatic nerve block, and obturator nerve block are associated with the most favorable clinical outcomes [3,4]. According to the latest evidence, the preoperative administration of a single-shot adductor canal block, in conjunction with intraoperative peri-articular local infiltration analgesia, is strongly recommended [3,4]. Despite the growing body of evidence supporting these approaches, the obturator nerve block, injection of the obturator nerve proximal to the bifurcation into anterior and posterior divisions in the plane between the pectineus and obturator externus muscles [5], remains a relatively underutilized procedure. This procedure may be considered in postoperative patients who, despite receiving multimodal analgesia, continue to experience moderate-to-severe anterior or posterior knee pain, without interfering with the surgical dressing or ambulation.

In this article, we describe the experience of three patients who, despite receiving an adductor canal block following total knee arthroplasty, continued to experience moderate-to-severe pain in the immediate postoperative period. In these cases, an obturator nerve block was administered as an adjunctive intervention, resulting in clinically significant analgesia and a marked improvement in pain control.

## Case presentation

All patients underwent total knee arthroplasty. In the operating room, spinal anesthesia was administered at the L3-L4 interspace in the sitting position, using 2 mL of 0.5% hyperbaric bupivacaine combined with 3 µg of sufentanil.

Case 1

A 68-year-old female patient, classified as American Society of Anesthesiologists (ASA) physical status III, with comorbidities including hypertension and diabetes, underwent total knee arthroplasty. At the conclusion of the surgical procedure, an ultrasound-guided adductor canal block was performed using 10 mL of 0.2% ropivacaine. Additionally, a catheter was positioned within the adductor canal for continuous infusion of 0.2% ropivacaine at a rate of 6 mL/h. Postoperatively, the patient was administered intravenous acetaminophen 1 g every eight hours and ketorolac 30 mg every eight hours for the first 24 hours.

Eighteen hours post-surgery, the perineural catheter placed in the adductor canal was inadvertently dislodged, causing leakage of the local anesthetic at the level of the surgical dressing. The patient reported pain in the posterior aspect of the knee, with a Numerical Rating Scale (NRS) [6] score of 6-7 (0-10 scale).

**Figure 1:**
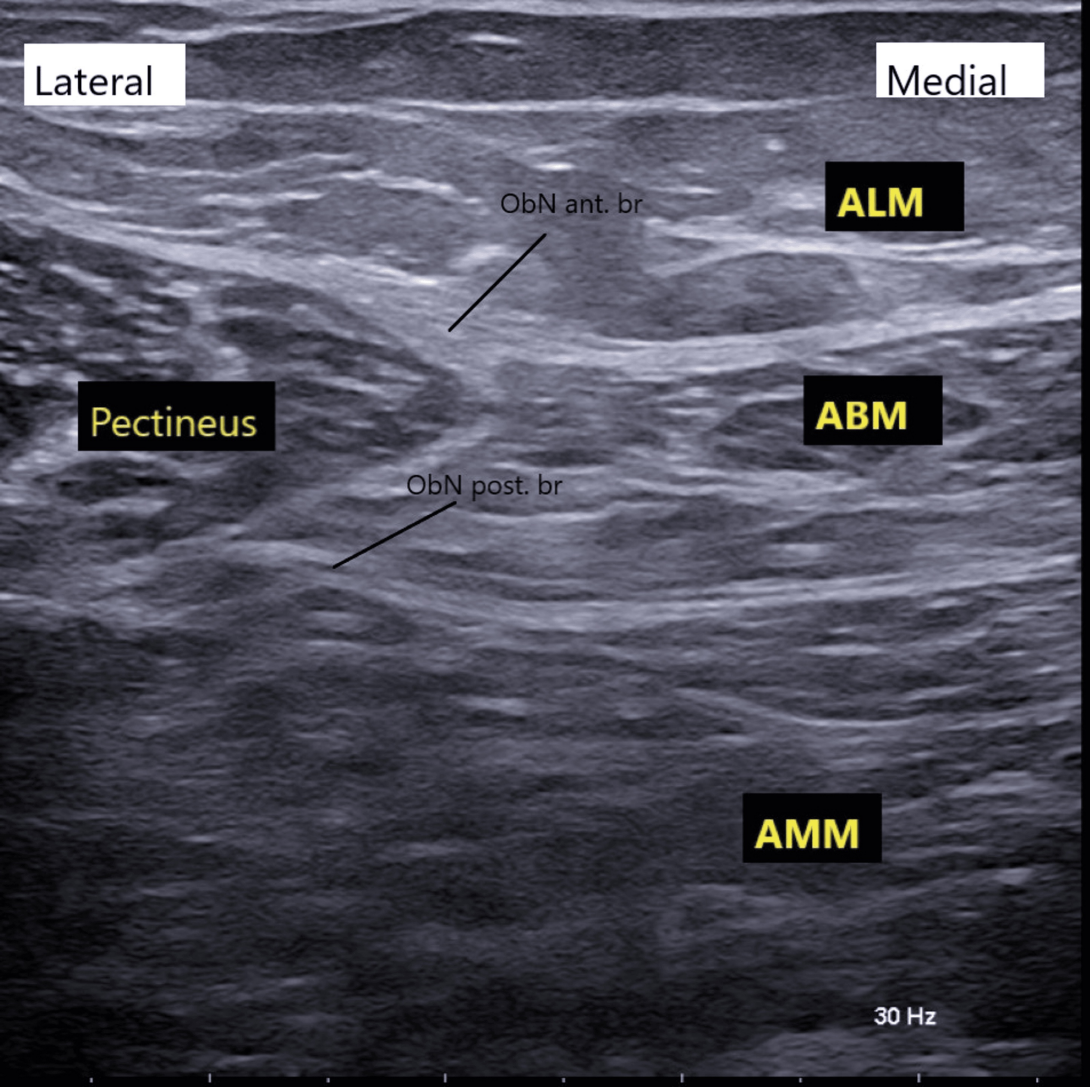
Ultrasonographic anatomy of the obturator nerve ALM, adductor longus muscle; ABM, adductor brevis muscle; AMM, adductor magnus muscle; Ob.N Ant.Br., obturator nerve anterior branch; Ob.N post.Br, obturator nerve posterior branch; Pectineus: pectineus muscle.

To manage the pain while maintaining the integrity of the surgical dressing and minimizing the risk of infection, an ultrasound-guided obturator nerve block was performed at a site remote from the surgical field. Strict aseptic precautions were followed throughout the procedure. The patient was positioned supine with the thigh slightly abducted. A high-frequency linear-array ultrasound transducer was placed transversely along the inguinal crease to identify the femoral neurovascular bundle. The probe was then advanced medially, approximately 1-2 cm distal to the inguinal ligament, until the pectineus and adductor muscle compartments and their respective fascial planes were clearly delineated, as illustrated in Figure 1.

**Figure 2:**
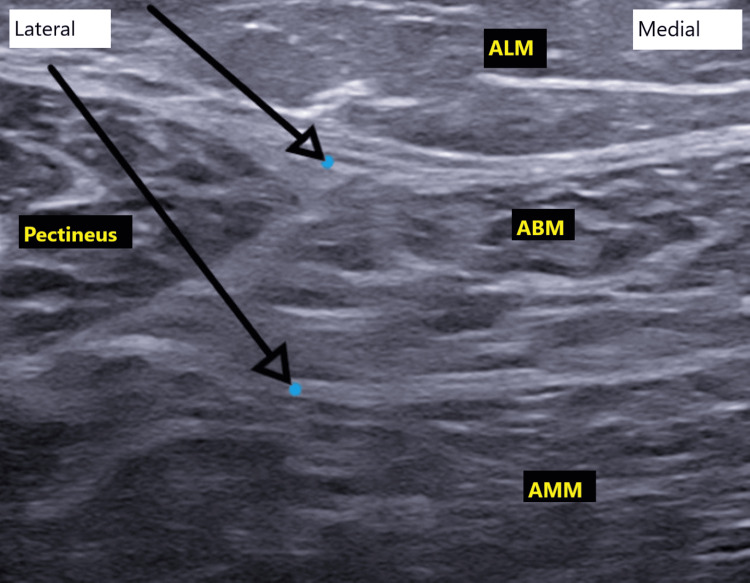
Ultrasound-guided obturator nerve block Ultrasound image showing the target points (blue dots) for final needle placement for local anesthetic injection between two muscle layers. ALM, adductor longus muscle; ABM, adductor brevis muscle; AMM, adductor magnus muscle; Pectineus: pectineus muscle.

The anterior branch of the obturator nerve is located within the fascial plane between the adductor brevis and either the pectineus or adductor longus muscles. The posterior branch lies within the fascial plane between the adductor brevis and adductor magnus muscles. Using an in-plane ultrasound-guided approach, a 22-G × 80 mm needle (Echoplex+, Vygon, Écouen, France) was advanced into the fascial plane between the adductor brevis and adductor magnus muscles. A total of 7 mL of 0.35% ropivacaine was administered to achieve blockade of the posterior branch of the obturator nerve. The needle was then gradually withdrawn until its tip was visualized within the fascial plane between the adductor longus and adductor brevis muscles, where an additional 7 mL of 0.35% ropivacaine was injected to block the anterior branch of the nerve, as shown in Figure 2.

Case 2

A 70-year-old patient, classified as American Society of Anesthesiologists (ASA) physical status II, with comorbidities including hypertension and obesity, underwent total knee arthroplasty. At the conclusion of the surgical procedure, a single-shot ultrasound-guided adductor canal block was performed using 15 mL of 0.5% ropivacaine.

Twenty-four hours postoperatively, the patient reported moderate-to-severe pain localized to the anteromedial and posterior aspects of the knee, refractory to non-steroidal anti-inflammatory drugs (NSAIDs) such as acetaminophen and ketorolac. The Numerical Rating Scale (NRS) score was 5-6 at rest and 7-8 during movement. In response, an ultrasound-guided obturator nerve block was performed, targeting both the anterior and posterior branches using the same technique and procedural approach described in Case 1.

Case 3

A 71-year-old male patient, classified as American Society of Anesthesiologists (ASA) physical status II, with a history of hypertension and a documented severe allergic reaction to NSAIDs, underwent left total knee arthroplasty. At the end of the procedure, a continuous ultrasound-guided adductor canal block was initiated. This included a 10 mL bolus of 0.2% ropivacaine followed by a continuous infusion of 0.2% ropivacaine at 6 mL/h. The patient also received intravenous acetaminophen 1 g every eight hours.

Twenty-six hours after surgery, the patient reported moderate pain localized to the anteromedial and posterior knee regions, with a Numerical Rating Scale (NRS) score of 5 at rest and 6-7 during movement. To manage the pain, an ultrasound-guided obturator nerve block was administered, targeting both the anterior and posterior branches using the same technique and anatomical landmarks previously described.

All patients received 8 mg of intravenous dexamethasone as an adjunct to prolong the analgesic effect [7]. Within 15 minutes of block administration, all patients reported complete resolution of pain with Numerical Rating Scale (NRS) 0 at rest and with movement. Importantly, no alterations in ambulation were observed, and the beneficial effect persisted for more than 24 hours.

## Discussion

Total knee arthroplasty (TKA) is a functional surgical procedure aimed at restoring joint mobility and improving quality of life, which requires early postoperative mobilization and rehabilitation to achieve optimal outcomes. However, early mobilization is often accompanied by significant postoperative pain, which may compromise recovery. Multimodal analgesic strategies incorporating the adductor canal block combined with the infiltration between the popliteal artery and the posterior capsule of the knee (IPACK) block have gained prominence due to their efficacy in providing targeted analgesia to both the anterior and posterior compartments of the knee, thereby facilitating improved pain control and rehabilitation [2,4,8]. Despite these benefits, potential complications and risks associated with the IPACK block, including peroneal nerve injury, intravascular injection, and the requirement for intra-articular infiltration prior to the procedure, represent notable limitations [9]. Furthermore, limited research has investigated the efficacy of the obturator nerve block as an alternative modality for postoperative analgesia following TKA [10].

Anatomically, within the popliteal fossa at the level of the femoral condyles, the posterior branch of the obturator nerve bifurcates into two to three terminal branches that traverse the medial aspect of the posterior knee capsule and form anastomoses with the popliteal nerve plexus. This anatomical configuration suggests that the obturator nerve contributes sensory innervation to both the anterior and posterior compartments of the knee. Accordingly, the obturator nerve block may be considered an alternative to other posterior compartment analgesic techniques, such as the IPACK block, potentially offering a reduced risk of infectious complications. In a randomized study by Runge et al. [11], the addition of an obturator nerve block to a femoral triangle block significantly reduced opioid consumption and postoperative pain after TKA compared to a single-shot femoral triangle block or local infiltration analgesia. The anterior branch of the obturator nerve forms an anastomosis with the saphenous nerve, mediating analgesic effects in the anteromedial compartment of the knee [12]. This anatomical interconnection allows for comparison of the obturator nerve block with other peripheral nerve blockade techniques, including the femoral nerve block and the adductor canal block.

Literature suggests that the obturator nerve block may serve as an adjunct to femoral or combined femoral and sciatic nerve blocks for postoperative analgesia following TKA [13], as well as to other peripheral nerve blocks [11]. The literature includes studies comparing the efficacy of the single adductor canal block (ACB) versus its combination with the obturator nerve block (ONB), such as the study by Dağdelen et al. [14]. Although the results did not reach statistical significance, a detailed analysis of the patients in Group II, who received both ACB and ONB, reported lower Visual Analogue Scale (VAS) scores during the first 24 postoperative hours and required fewer opioids compared to those who received single ACB. The results obtained in our three patients were similarly encouraging, suggesting that the obturator nerve block, when used in conjunction with the adductor canal block, may provide effective analgesia without impairing ambulation, thus facilitating early postoperative rehabilitation.

## Conclusions

Postoperative pain management remains a fundamental and challenging aspect of perioperative care in TKA. Peripheral nerve blocks, administered as continuous infusions or single-shot injections of local anesthetics, represent well-established and evidence-based techniques that provide effective analgesia and contribute to improved functional recovery. The choice of analgesic strategy should be individualized, taking into account patient characteristics, comorbidities, and the anticipated trajectory of postoperative recovery. Based on our clinical experience, the obturator nerve block provides effective postoperative analgesia without compromising early ambulation and may serve as an adjunct or alternative to more commonly employed techniques in the context of TKA. Nevertheless, further studies with larger patient cohorts are warranted to confirm its efficacy and delineate its role within standardized analgesic protocols.

## References

[1] Lee JH, Choi YS (2019). Nerve blocks for optimal postoperative analgesia after total knee arthroplasty. Anesth Pain Med.

[2] Lavand'homme PM, Kehlet H, Rawal N, Joshi GP (2022). Pain management after total knee arthroplasty: PROcedure SPEcific Postoperative Pain ManagemenT recommendations. Eur J Anaesthesiol.

[3] Qin L, You D, Zhao G, Li L, Zhao S (2021). A comparison of analgesic techniques for total knee arthroplasty: a network meta-analysis. J Clin Anesth.

[4] El-Boghdadly K, Albrecht E, Wolmarans M (2024). Standardizing nomenclature in regional anesthesia: an ASRA-ESRA Delphi consensus study of upper and lower limb nerve blocks. Reg Anesth Pain Med.

[5] Albrecht E, Renard Y, Desai N (2024). Intravenous versus perineural dexamethasone to prolong analgesia after interscalene brachial plexus block: a systematic review with meta-analysis and trial sequential analysis. Br J Anaesth.

[6] Downie WW, Leatham PA, Rhind VM, Wright V, Branco JA, Anderson JA (1978). Studies with pain rating scales. Ann Rheum Dis.

[7] Bendtsen TF, Moriggl B, Chan V, Børglum J (2016). The optimal analgesic block for total knee arthroplasty. Reg Anesth Pain Med.

[8] El-Emam EM, El Motlb EA (2020). Ultrasound-guided adductor canal block versus combined adductor canal and infiltration between the popliteal artery and the posterior capsule of the knee block for osteoarthritis knee pain. Anesth Essays Res.

[9] Biehl M, Wild L, Waldman K, Haq F, Easteal RA, Sawhney M (2020). The safety and efficacy of the IPACK block in primary total knee arthroplasty: a retrospective chart review. Can J Anaesth.

[10] Wang F, Wu J, Wu Y, Han X, Dai H, Chen Q (2024). Different peripheral nerve blocks for patients undergoing total knee arthroplasty: a network meta-analysis of randomized controlled trials. Arch Orthop Trauma Surg.

[11] Runge C, Børglum J, Jensen JM (2016). The analgesic effect of obturator nerve block added to a femoral triangle block after total knee arthroplasty: a randomized controlled trial. Reg Anesth Pain Med.

[12] Tran J, Peng PW, Gofeld M, Chan V, Agur AM (2019). Anatomical study of the innervation of posterior knee joint capsule: implication for image-guided intervention. Reg Anesth Pain Med.

[13] Terkawi AS, Mavridis D, Sessler DI (2017). Pain management modalities after total knee arthroplasty: a network meta-analysis of 170 randomized controlled trials. Anesthesiology.

[14] Dağdelen MS, Abut FY, Erden V, Seven S (2019). Postoperative analgesia after combined obturator nerve and adductor canal block in total knee arthroplasty. Int J Anesthetic Anesthesiol.

